# Pharmacokinetic Prediction and Cytotoxicity of New Quercetin Derivatives

**DOI:** 10.1002/cbdv.202500119

**Published:** 2025-05-09

**Authors:** Michele Goulart dos Santos, Marie Demonceaux, Lucia Emanueli Schimith, Marine Goux, Claude Solleux, Ana Luiza Muccillo‐Baisch, Bruno Dutra Arbo, Corinne Andre‐Miral, Mariana Appel Hort

**Affiliations:** ^1^ Programa de Pós‐Graduação em Ciências Fisiológicas, Instituto de Ciências Biológicas, Universidade Federal do Rio Grande Rio Grande Rio Grande do Sul Brazil; ^2^ Unit at the Biological Sciences at Biotechnologies Nantes University Nantes France; ^3^ Programa de Pós‐Graduação em Ciências da Saúde, Faculdade de Medicina, Universidade Federal do Rio Grande Rio Grande Rio Grande do Sul Brazil; ^4^ Departamento de Farmacologia Instituto de Ciências Básicas da Saúde, Universidade Federal do Rio Grande do Sul Porto Alegre Rio Grande do Sul Brazil

**Keywords:** ADME analysis, cytotoxicity, flavonoid, physicochemical properties, transglucosylation

## Abstract

Quercetin (QUE) possesses various pharmacological properties; however, its low bioavailability and solubility hinder its beneficial effects. Enzymatic glycosylation has been explored to improve these aspects. In the present study, we used a sucrose phosphorylase variant to catalyze the regioselective transglucosylation of QUE, predicted the pharmacokinetic properties and toxicity of these molecules using in silico tools, and evaluated their cytotoxicity compared to the original molecule and a β‐glucosylated derivative of QUE. Three α‐glucosylated derivatives were obtained, which demonstrated improved pharmacokinetics, including a higher volume of distribution and lower clearance rate, with minimal likelihood of cytochrome P450 enzyme inhibition compared to QUE. QUE and the β‐glucosylated derivative exhibited cytotoxicity in both cell types evaluated, whereas their α‐glucosylated derivatives were nontoxic. The results presented provide an insight into the predicted behavior of these molecules in the body and, combined with cytotoxicity evaluation, will serve as a foundation for investigating the biological effects and mechanisms of action of these new molecules.

## Graphical abstract

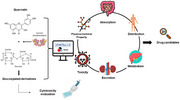

## 1 Introduction

Quercetin (QUE) (3,3′,4′,5,7‐pentahydroxyflavone) is a flavonoid found in large quantities in vegetables and fruits such as almonds, capers, coriander, apples, and onions [1]. It is one of the most important plant molecules, with well‐described pharmacological properties, including antioxidant [2], anti‐inflammatory [3], antiviral [4], anticancer [5], and neuroprotective activity [6]. QUE is considered a lipophilic molecule; thus, it is presumed to have the ability to cross biological membranes through simple diffusion. However, it has low bioavailability, low aqueous solubility, and instability [7], factors that limit its absorption in the digestive tract. Additionally, its chemical form also influences absorption, and studies have shown that the aglycone form of QUE, commonly found in supplements, is less bioavailable than its glycosides, which occur naturally in foods [8, 9, 10]. This structural difference affects not only bioavailability but also other pharmacokinetic parameters, as hydrolysis of the glycosidic moiety is essential for the molecule to be absorbed in the intestine [11, 12]. Given these limitations, several studies have been conducted to modify its structure in order to optimize its therapeutic properties [13].

**FIGURE 1:**
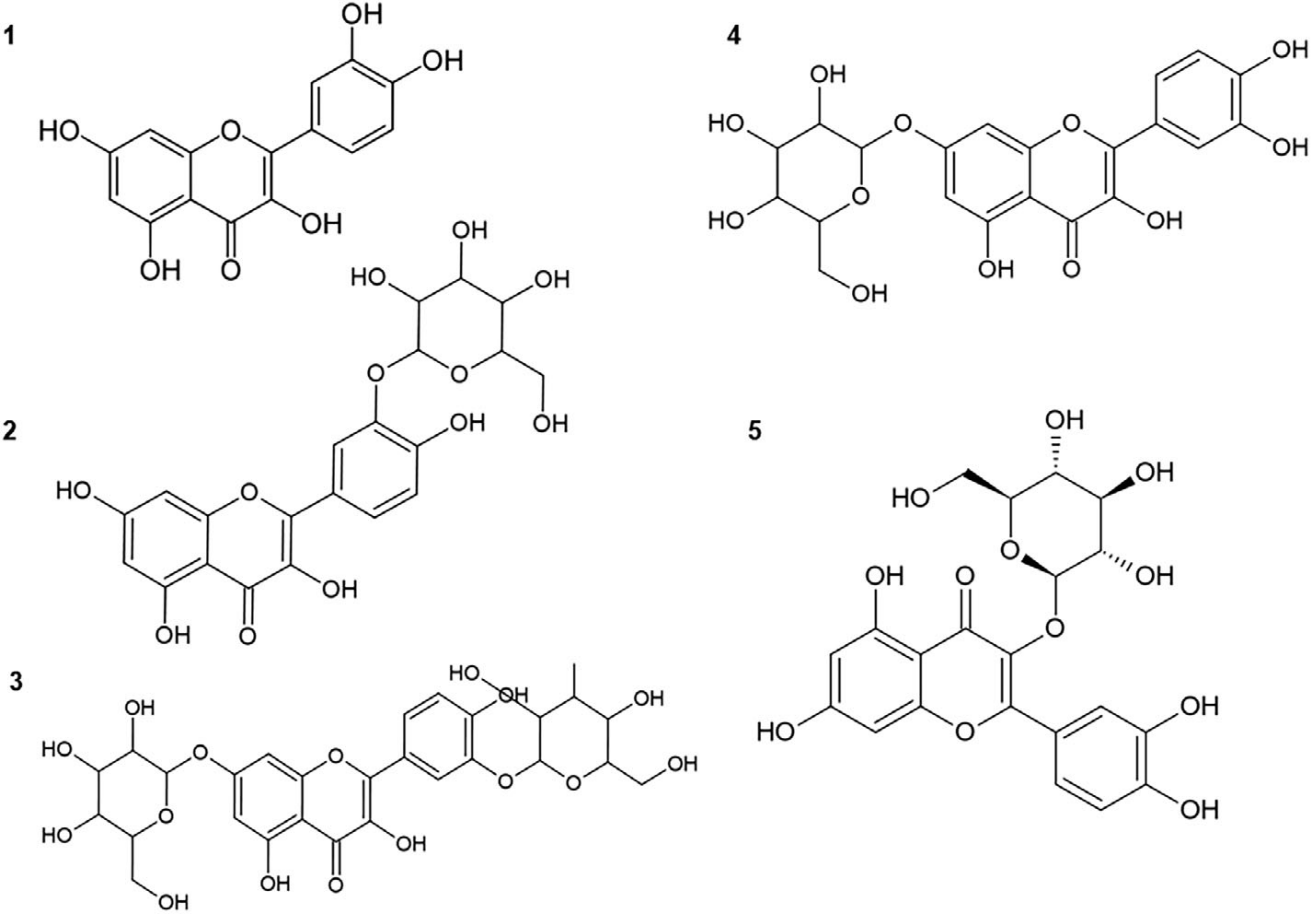
Chemical structure of (1) quercetin; (2) quercetin‐3′‐α‐d‐glucoside; (3) quercetin‐3′,7‐α‐d‐diglucoside; (4) quercetin‐7‐α‐d‐glucoside; and (5) quercetin 3‐β‐d‐glucoside.

The chemical structure of QUE contains five hydroxyl groups at positions 3, 5, 7, 3′, and 4′ (Figure 1), which can be glycosylated to form various glycosides [14]. Glycosylation refers to the addition of any sugar to a flavonoid structure, whereas glucosylation is specific to the addition of glucose [15]. Plants have the ability to produce a variety of glycosylated flavonoids; for example, QUE can be found bound to different sugars, such as glucose, galactose, rhamnose, and rutinose, among others [8]. However, glycosides are generally produced in small quantities and as mixtures, which makes the extraction and purification process complex [16].

In this regard, enzymatic glycosylation of flavonoids has attracted considerable interest. The glycosylation process basically involves adding a sugar moiety to the molecule, thereby producing derivatives with multiple sugar units at different hydroxyl groups, which not only create new metabolites but also enhance the activity and functions of the original molecule [15]. This structural optimization is an important tool for modifying the physicochemical properties of the molecule and can be achieved through microorganisms designed to express biocatalysts that carry out the desired modification. Among the existing biocatalysts, sucrose phosphorylases (SPs) stand out, which are particularly interesting due to their substrate (sucrose) and their ability to use different co‐substrates, as well as the presence of thermostable homologs. These enzymes essentially transfer glucose from sucrose to an acceptor to produce α‐glucosylated products. Only a few studies report the use of SP for the α‐glucosylation of flavonoids [17, 18, 19].

The thermostable SP from *Bifidobacterium adolescentis* (BaSP) and its variants have been investigated for their ability to produce rare disaccharides through biocatalytic synthesis [20, 21] and to perform α‐glycosylation of polyphenols [17, 18, 22]. The presence of the sugar portion in the structure of the original molecule can give it better water solubility, greater stability, and, potentially, bioavailability, making it suitable for applications in various areas, such as health [23].

In the present study, we investigated the ability of the variant Q345F of BaSP to catalyze the regioselective transglucosylation of QUE, a variant that has already been investigated in the process of transglucosylation of other polyphenols such as resveratrol and catechin [18, 22]. Additionally, we employed computational approaches to predict the pharmacokinetic properties and toxicity of these molecules and determine how the addition of sugar groups could modify these parameters. Glucosylation is a promising strategy for modulating the physicochemical properties of drugs, including absorption in the gastrointestinal tract and other factors such as half‐life, a characteristic that can prolong therapeutic activity, as well as distribution in tissues, particularly in the nervous system [24]. Glucosylation may influence the molecule's ability to cross biological barriers, such as the blood–brain barrier (BBB), which regulates the entry of substances from the blood into the brain [25]. In this context, we also investigated the cytotoxicity of the QUE and its derivatives using brain‐derived cell lines.

## 2 Materials and Methods

### 2.1 Production, Purification, and Characterization of the BaSP Q345F Enzyme

The BaSP Q345F enzyme was obtained as previously described [22]. Briefly, *Escherichia coli* BL21(DE3), transformed with a pET28b vector containing the enzyme gene, was incubated in 5 mL of LB medium supplemented with kanamycin (25 µg/mL) and glucose (20%, v/v) overnight at 37°C. The following day, 2 mL of the culture was incubated in 200 mL of auto‐inducible medium supplemented with kanamycin (25 µg/mL) at 25°C for 24 h. The cultures were centrifuged (Thermo Scientific, Sorvall RC6 plus, SLC 4000 rotor, 30 min, 4150 g, 19°C), and the supernatant was collected for two purification steps on nickel Ni‐NTA resin (Protino Ni‐NTA, Macherey‐Nagel) and elution with NPI buffer (50 mM NaH_2_PO4, 300 mM NaCl, 250 mM imidazole‐HCl, pH 8.0) after a series of washes to obtain a good purification of the enzyme. The purification efficiency was identified using SDS–PAGE gel electrophoresis, and the proteins were quantified (NanoDrop 1000, Thermo Scientific). The evaluation of BaSP Q345F thermostability and kinetics was done as described in Demonceaux et al. [22].

### 2.2 Transglucosylation Reaction

The reactions were carried out in a solution of 3‐morpholinopropane‐1‐sulfonic acid (MOPS)‐NaOH (50 mM, pH 8.0), containing QUE (10 mM), 30% DMSO, 80 mM sucrose, and incubated with a final concentration of 10 µM of purified enzyme at 37°C for BaSP Q345F with agitation for 24 h.

### 2.3 Purification and Identification of Glucosylated Quercetin Derivatives

The composition of the reaction mixture was characterized by analytical HPLC at 280 nm using a C‐18 column (Interchim, 5 µm, 250 × 4.6 mm^2^, US5C18HQ‐250/046) with an isocratic flow of 80% H_2_O (v/v), 0.1% trifluoroacetic acid (TFA) (v/v), and 20% acetonitrile (v/v), 0.1% TFA (v/v) for 20 min, as previously described [18]. Compound concentrations were calculated from the area under the curves (AUCs). In addition, after 24 h of incubation, the reaction medium was centrifuged again (12 000 g, 20 min), and the remaining supernatant was purified by preparative HPLC at 280 nm using a C‐18 column (Interchim, 5 µm, 250 × 21.2 mm^2^, US5C18HQ‐250/212) with a gradient system (solvent A: H_2_O, TFA 0.1%; solvent B: Acetonitrile, TFA 0.1%; t0 min = 70/30, t10 min = 70/30, t70 min = 10/90). The injection volume was 2 mL containing 10 mM quercetin. Using the conversion yield, the amount of each product obtained can be calculated. The products were identified by mass spectroscopy (H mode, Waters, high‐resolution UPLC‐MS2) and 1H and 13C NMR in MeOD or DMSO‐d6 (400 Hz, 256 scans) as previously described by Kraus [17, 26], for the characterization of the QUE derivatives (QUE‐3′, QUE‐7, and QUE‐3′,7) (Supporting Information).

### 2.4 In Silico Prediction of Pharmacokinetic Properties of Quercetin and Derivatives

After identifying the products obtained from the enzymatic transglucosylation reaction, their physicochemical properties were compared using two platforms that determine pharmacokinetic and toxicological characteristics, namely, SwissADME (http://www.swissadme.ch/) and ADMETlab (https://admetmesh.scbdd.com/). The sdf files containing the molecular structures of the molecules were uploaded to the platforms, and subsequently, absorption, distribution, metabolism, excretion, and toxicity data were extracted from ADMETlab 2.0. Bioavailability radar and drug‐likeness, based on the Lipinski rule, were obtained from SwissADME.

### 2.5 Evaluation of the In Vitro Toxicity of Quercetin and Derivatives

#### 2.5.1 Cell Culture

The rat astroglioma cell line (C6) and the human neuroblastoma cell line (SH‐SY5Y) were obtained from the Rio de Janeiro Cell Bank (Rio de Janeiro, Brazil). The cell cultures were maintained in DMEM culture medium for C6 cells and DMEM F12 for SH‐SY5Y cells, both supplemented with 10% FBS and 1% penicillin‐streptomycin and amphotericin B. The cultures were maintained in a humidified atmosphere at 37°C containing 5% CO_2_. The cells were cultured until they reached 70%–80% confluence and used up to the passage limit of 30.

#### 2.5.2 Cell Viability Assay

The effects of QUE and its derivatives on cell viability were analyzed using the MTT (3‐(4,5‐dimethylthiazol‐2‐yl)‐2,5‐diphenyltetrazolium bromide) reduction assay, as described by Mosmann [27]. Briefly, cells were seeded in 96‐well plates, with C6 cells cultured at a density of 6 × 10^3^ cells per well and SH‐SY5Y cells cultured at a density of 1.8 × 10^4^ cells per well. After 24 h, cells were exposed to concentrations of 10, 25, 50, and 100 µM of QUE or derivatives, or vehicle (0.6% DMSO for the highest concentration of derivatives and 0.2% DMSO for the highest concentration of QUE) for an additional 24 h. QUE (50 mM) and derivatives (15 mM) stock solutions were prepared in DMSO, and working solutions were prepared in serum‐free culture medium. Following the treatment period, cells were incubated with MTT (0.5 mg/mL) for 1 h for C6 cells and 1 h and 30 min for SH‐SY5Y cells, both at 37°C, followed by the addition of 100 µL of DMSO. Absorbance was read at 550 nm using a microplate reader (ELx800 Microplate Reader, Biotek Instruments, Vermont, USA). Treatments were performed in triplicate in three independent experiments. Data on cell viability were expressed as a percentage of control (untreated cells).

### 2.6 Statistical Analysis

Statistical analyses were performed using GraphPad Prism version 9 for Windows (GraphPad Software, USA). The data result was checked for normal distribution with Shapiro–Wilk and Kolmogorov–Smirnov. Data were represented as mean and standard error of the mean and analyzed using one‐way ANOVA followed by Tukey's post hoc test. The level of significance was set at *p* ≤ 0.05.

## 3 Results and Discussion

### 3.1 Obtaining and Chemical Characterization of Quercetin Glucoside Derivatives

**FIGURE 2:**
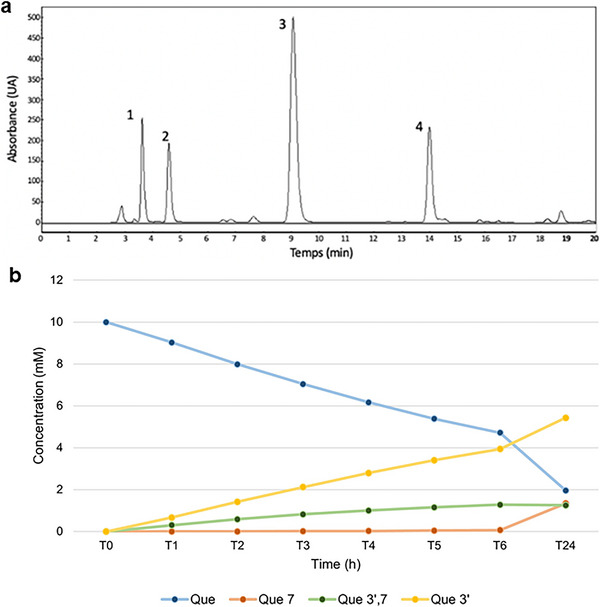
(a) Analytical HPLC chromatogram of a reaction medium of quercetin with BaSP Q345F over 24 h: (1) quercetin‐3′,7‐*O*‐α‐d‐diglucoside; (2) quercetin‐7‐*O*‐α‐d‐glucoside; (3) quercetin‐3′‐*O*‐α‐d‐glucoside; and (4) quercetin. (b) Kinetics of formation of the products formed with BaSP Q345F over 24 h obtained with analytical HPLC.

The enzymatic transglucosylation of QUE was performed using BaSP Q345F as catalyst and QUE and sucrose as acceptor and donor, respectively. The glucosylated products were isolated by a reverse‐phase HPLC C‐18 (Figure 2), and their structures were confirmed by NMR ^1^H, ^13^C, and MS (Supporting Information Figures S1‐S4). We obtained quercetin‐3′‐*O*‐α‐d‐glucoside as majority product (QUE‐3′, 68%, retention time 9–10 min), as well as quercetin‐7‐*O*‐α‐d‐glucoside (QUE‐7, 15%, retention time 4–5 min) and quercetin‐3′,7‐*O*‐α‐d‐diglucoside (QUE‐3′,7, 17%, retention time 3–4 min) as secondary products. For these three compounds, presence of mono‐ or diglucosylation was confirmed by the peaks integration between 3.4 and 4.0 ppm and by the peaks corresponding to the anomeric protons at 5.4 and/or 5.7 ppm with coupling constant J of 3.3–3.8 Hz < 5 Hz, validating an α‐conformation (Table S1). The addition of a glucose moiety on ring A and/or B of QUE resulted in a deshielding of the protons of these rings. The use of the BaSP Q345F mutant resulted only in the synthesis of these derivatives, as the regioselectivity of the reaction depends on interactions within the enzyme's catalytic site. The three glucosylated positions are those that allow for the greatest stabilization of quercetin within the catalytic site, as described by Kraus et al. [17]. The Position 3 on ring C is not accessible to SPs, and glucosylation at positions 4′ or 5 was not observed with this enzyme mutant.

Previous molecular docking analyses by Kraus et al. (2016) [17] elucidated the mechanism responsible for the altered catalytic properties of the Q345F variant. Crystals of BaSP Q345F were grown in the presence of sucrose, and a crystal structure complexed with glucose (a hydrolysis product) at 2.7 Å resolution was solved (PDB ID: 5C8B). The most pronounced differences between the crystal structure of the Q345F variant and the wild‐type enzyme are observed in the region encompassing residues 86–166 (domain B), which is displaced relative to the rest of the protein. Rearrangements in the A and B loops, as well as in β‐sheet A, contribute to the altered catalytic properties of BaSP Q345F.

The yields obtained are in agreement with those obtained by Kraus et al. [26], and the same three products were synthesized with a ratio of 41:11:48 for other flavonoids. The differences of yields can be explained by our optimization of the protocol for the enzymatic reaction as previously described [22].

All the derivatives formed by the enzyme have linked orientation of the glucosidic group in the α conformation. This can affect the solubility, stability, and biological activity of the resulting derivatives [28]. Under natural conditions, β‐glucosides are the predominant derivatives [29], and the presence of QUE α‐glucosides in nature is less common and not well reported in the literature, making the synthesis and study of α‐conformations particularly significant. Understanding the properties and behaviors of α conformations allows researchers to explore novel chemical spaces and potentially uncover unique biological activities that may not be present in naturally occurring beta conformations.

QUE has numerous other glucosylated derivatives in the β position. Isoquercetin, for example, is a compound that has glucose attached to the 3‐OH group (quercetin‐3‐β‐d‐glucoside (QUE‐3β)). Furthermore, when galactose binds to quercetin's‐3‐OH, hyperoside (quercetin‐3‐*O*‐galactoside) is formed. Similarly, rhamnosyl can also bind to 3‐OH or 7‐OH, resulting in the synthesis of quercetin‐3‐*O*‐ramnoside or quercetin‐7‐*O*‐ramnoside, respectively. Another QUE derivative with two sugars present in its structure and very well documented in the literature is rutin, with the hydroxyl group at the C‐3 position replaced by glucose and rhamnose sugar groups [30]. Similarly, avicularin contains arabinofuranose linked to quercetin‐3‐OH [29]. In addition, some derivatives of QUE can have more than two sugar residues, such as enzymatically modified isoquercitrin (EMIQ), which can have up to 10 glucose residues linked to the 3‐OH of QUE [31].

Given that previous studies suggest that the biological activities of QUE and its derivatives can vary significantly depending on structural modifications, such as the α or β glucoside conformations [14, 32], we decided to investigate these differences comparatively. Although β‐glucosylated derivatives, such as isoquercetin, have been extensively studied, α‐glucosylated derivatives remain unexplored regarding their biological activity. Therefore, we conducted an in silico analysis and cytotoxicity tests to compare the synthesized α derivatives with a β derivative, aiming to better understand how glucoside conformation influences molecular interactions with biological targets and the potential pharmacological effects.

### 3.2 In Silico Pharmacokinetic Profile (ADMET)

The application of in silico tools is essential for predicting and understanding potential alterations induced by molecules in biological systems, as they employ advanced computational methods to simulate molecular interactions, protein structures, and even metabolic networks. These tools not only aid in predicting changes but also in designing more effective and safer therapeutic interventions. The results obtained from the ADMETlab and SwissADME analyses of the pharmacokinetic parameters of QUE and its derivatives (absorption, distribution, metabolism, and excretion) are summarized in Table 1.

**TABLE 1:** Pharmacokinetic parameters (ADME) prediction.

	Molecules	Absorption				
N°		LogP (0–3)	PSA (0–140)	Pgp‐inhibitor (0–1)	Pgp‐substrate (0–1)	GI absorption
1	Quercetin	2.155	131.36	0.004	0.005	High
2	Quercetin‐3′‐α‐d‐glucoside	−0.133	210.51	0.004	0.75	Low
3	Quercetin‐3,7‐α‐d‐diglucoside	−1.335	289.66	0.001	0.988	Low
4	Quercetin‐7‐α‐d‐glucoside	−0.1	210.51	0.004	0.681	Low
5	Quercetin‐3‐β‐d‐glucoside	−0.0	210.51	0.009	0.447	Low

		Distribution		
	Molecules	VD (0.04–20 L/kg)	BBB penetration (0–0.3)	PPB (<90%)
1	Quercetin	0.579	0.008	95.49%
2	Quercetin‐3′‐α‐d‐glucoside	0.895	0.025	88.89%
3	Quercetin‐3′,7‐α‐d‐diglucoside	0.661	0.259	73.26%
4	Quercetin‐7‐α‐d‐glucoside	0.871	0.021	88.91%
5	Quercetin‐3‐β‐d‐glucoside	0.872	0.027	88.05%

	Metabolism					
		Metabolic enzymes (S: substrate; I: inhibitor) (Category 0: non‐substrate/non‐inhibitor; Category 1: substrate/inhibitor)				
	Molecules	CYP1A2	CYP3A4	CYP2C9	CYP2C19	CYP2D6
1	Quercetin	S: 0.115 I: 0.943	S: 0.046 I: 0.348	S: 0.643 I: 0.598	S: 0.041 I: 0.053	S: 0.205 I: 0.411
2	Quercetin‐3′‐α‐d‐glucoside	S: 0.035 I: 0.073	S: 0.01 I: 0.07	S: 0.205 I: 0.012	S: 0.047 I: 0.013	S: 0.167 I: 0.051
3	Quercetin‐3′,7‐α‐d‐diglucoside	S: 0.012 I: 0.017	S: 0.001 I: 0.006	S: 0.089 I: 0.0	S: 0.053 I: 0.003	S: 0.142 I: 0.002
4	Quercetin‐7‐α‐d‐glucoside	S: 0.033 I: 0.079	S: 0.009 I: 0.046	S: 0.13 I: 0.014	S: 0.047 I: 0.012	S: 0.163 I: 0.039
5	Quercetin‐3‐β‐d‐glucoside	S: 0.036 I: 0.145	S: 0.009 I: 0.072	S: 0.273 I: 0.019	S: 0.046 I: 0.017	S: 0.168 I: 0.086

		Excretion	
	Molecules	Half‐life time (0–0.3: T1/2 > 3 h; 0.3–0.7: moderate; 0.7–1.0: T1/2 < 3 h)	Clearance (mL/min/kg) (>15: high; 5–15: moderate; <5: low)
1	Quercetin	0.929	8.284
2	Quercetin‐3′‐α‐d‐glucoside	0.818	3.349
3	Quercetin‐3′,7‐α‐d‐diglucoside	0.452	1.325
4	Quercetin‐7‐α‐d‐glucoside	0.795	3.833
5	Quercetin‐3‐β‐d‐glucoside	0.898	4.956

*Note*: The values in parentheses indicate the range considered ideal.

Abbreviations: BBB, blood–brain barrier; GI, gastrointestinal absorption; LogP, octanol‐water partition coefficient; P‐gP, P‐glycoprotein; PPB, Binding to plasma proteins (PPB); PSA, Polar surface area; VD, Volume of distribution.

The results obtained for the octanol‐water partition coefficient (LogP) showed that QUE has a high lipophilicity and a polar surface area (PSA) within the ideal levels (0–140), so it is assumed that it can cross biological membranes by passive diffusion [33]. The glucosylated derivatives showed low lipophilicity and consequently a higher PSA, especially QUE‐3′,7 which showed the highest PSA value (289.66), exceeding the ideal range (0–140), indicating that these molecules have low permeability in the lipid bilayer. It is assumed that these derivatives depend on specific transporters that can facilitate the absorption of QUE glucosides, such as the sodium‐dependent glucose transporter‐1 (SGLT‐1) and glucose transporter 2 (GLUT‐2), that are transporters capable of contributing to greater intestinal uptake of glycosides [34]. The results for QUE‐7α and QUE‐3β showed the same PSA value, indicating no difference in this parameter between the alpha and beta conformations.

Before being absorbed, QUE glucosides must be deglucosylated into aglycone by enzymes such as lactase‐phlorizin hydrolase (LPH) and/or cytosolic β‐glucosidases (CBG) present in the brush border of enterocytes [35]. This process is fundamental to increase the absorption of these derivatives in the intestine, which contributes to enhancing their plasma concentration and improving their bioavailability [35]. Considering that these enzymes are specific to glucose, the glucosylated derivatives analyzed here are absorbed more quickly than other QUE glycosides that have other types of sugars in their structure [36]. However, the α or β conformation may influence the absorption of these glucosylated derivatives due to the distinct interactions that each conformation establishes with intestinal transporters and other proteins involved in the absorption process, although in silico analyses showed similar values for the QUE‐3β derivative with QUE‐7α. Studies indicate that β‐glucosylated derivatives, such as QUE‐3β, tend to be better recognized by transporters than α‐glucosylated ones [37, 38], potentially leading to more efficient absorption for the β conformation. Nonetheless, specific experimental data on α‐glucosylated derivatives remain limited and warrant further investigation.

According to Lipinski's rule, in addition to LogP, molecular weight (MW) should be less than 500 Da, the number of nitrogen‐bonded hydrogen donors (HBD) should be less than or equal to 5 nOHNH, and the number of hydrogen acceptors (HBA) should be less than or equal to 10 nON. QUE did not present any violation of this rule; however, the QUE‐3′α, QUE‐7α, and QUE‐3β derivatives showed two violations of the rule (NorO > 5 and NHorOH > 10). For QUE‐3′,7, there were three violations of the rule, the two mentioned above and the MW of more than 500 Da (624 g/mol).

The Abbot Bioavailability Score is a parameter that seeks to predict the likelihood of a compound having at least 10% oral bioavailability in rats or measurable permeability in Caco‐2 cells. This score considers the molecule's total charge, PSA, and Lipinski rule violations, defining four classes of compounds with probabilities of 11%, 17%, 56%, or 85%. Derivatives concentrate in the second class, whereas QUE falls into the third.

Despite in silico predictions suggesting higher bioavailability for QUE, several preclinical studies have demonstrated that glucosides of QUE exhibit greater bioavailability compared to the aglycone form. Research conducted on dogs revealed that the QUE‐3β derivative, which includes a glucose molecule attached at Position 3 of QUE, contributed to a threefold increase in the bioavailability of QUE [10]; however, the potential in vivo effects of the molecule still need to be further investigated in other species, especially in humans, as observed in the study by Hollman et al. [39], QUE aglycone in capsule form showed lower absorption than QUE glycosides present in fried onions, a food that predominantly contains the glycosylated form. A more recent human study also demonstrated that onion soup has higher bioavailability than isolated supplements, reinforcing the idea that a glycoside‐rich food matrix plays a crucial role in QUE absorption [40].

Another study comparing the bioavailability of QUE and isoquercitrin on rats indicated consistently higher levels of metabolites in various tissues and plasma samples in rats fed isoquercitrin, suggesting isoquercitrin's potential to enhance QUE bioavailability [41]. Therefore, glucosylated derivatives may serve as precursors to the aglycone, potentially enhancing the plasma bioavailability of the molecule, as these glucosides exhibit greater water solubility than the aglycone form, evidenced by their lower octanol‐water partition coefficient [42]. Furthermore, as previously mentioned, glucosylated derivatives are likely absorbed in the intestine via glucose transporters such as SGLT‐1, a pathway not utilized by aglycone QUE [43]. This transporter‐mediated route may facilitate greater intestinal uptake of the glycoside, contributing to its potentially higher bioavailability compared to the free form.

Expanding on these data, the probability of molecules crossing the BBB was also assessed, as represented in Table 1. QUE and its derivatives fell within the optimal range regarding the probability of penetrating the BBB, with QUE showing the highest probability and derivative QUE‐3′,7 the lowest probability compared to others, but still within the optimal range. The ability to penetrate the BBB is the initial requirement for treating central nervous system (CNS) diseases [44], including neurodegenerative diseases such as Alzheimer's and Parkinson's, psychiatric disorders like depression and schizophrenia, inflammatory and autoimmune diseases, brain tumors, and cerebrovascular disorders [45, 46, 47]. This barrier possesses a distinctive structure, characterized by tight junctions, overexpression of enzymes, and efflux proteins, which collectively inhibit the passage of the majority of drugs [48]. Glucosylated derivatives of QUE demonstrated a lower probability of crossing the BBB when compared to QUE, and there were no significant differences between the α monoglucosylated derivatives and the β derivative. These data are limited to the passive diffusion process only and take into account other parameters such as the lipophilic nature of the molecule. However, among the various enzymes overexpressed in the BBB are the GLUT‐1 transporters, responsible for facilitating the transport of essential nutrients to the brain, such as glucose [49]. Due to the efficiency of this transporter, GLUT‐1 has emerged as a significant target in drug development strategies aimed at the brain. It is recognized that the number of sugar moieties can influence the solubility and targeting capacity of molecules to the brain [49].

**FIGURE 3:**
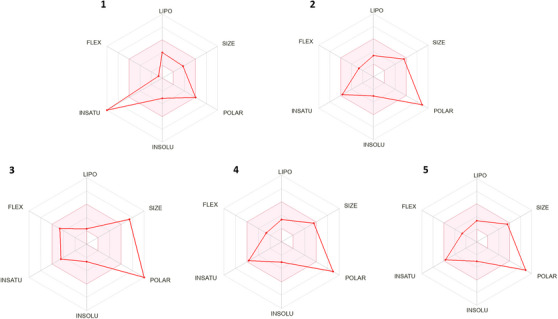
Bioavailability radar chart from Swiss ADME online web tool for compounds (1) quercetin; (2) quercetin‐3′‐α‐d‐glucoside; (3) quercetin‐3′,7‐α‐d‐diglucoside; (4) quercetin‐7‐α‐d‐glucoside; and (5) quercetin 3‐β‐d‐glucoside. The pink area represents the optimal range for each property.

The bioavailability radar analysis on the SwissADME platform consists of a graphical representation that combines different parameters related to the oral absorption of a compound (Figure 3). It is calculated on the basis of six physicochemical characteristics of the compound: molecular size, lipophilicity, polarity, solubility, flexibility, and saturation. The aim is to provide a rapid visual assessment of the compound's possible oral absorption. To be estimated as drug‐like, the red line of the study molecules must be completely included in the pink area. Any deviation represents a suboptimal physicochemical property for oral bioavailability [50]. For example, for saturation, the proportion of sp^3^ hybridized carbons in relation to the total carbons in the molecule (Csp^3^ fraction) must be at least 0.25. For size, the MW must vary between 150 and 500 g/mol. For polarity, the TPSA must be between 20 and 130 Å^2^. For solubility, the log S (calculated using the ESOL model) must not exceed 6. As for lipophilicity, XLOGP3 must be between −0.7 and +6.0. With regard to flexibility, the molecule must not have more than nine rotatable bonds.

After inputting the compounds into the SwissADME platform, the predicted physicochemical properties mentioned were assessed. QUE apparently did not adequately meet the flexibility and unsaturation criteria compared to its derivatives, which slightly encompassed these criteria more, with QUE showing unsaturation below the required minimum. Regarding polarity, QUE is at the maximum established value limit of 130 Å; however, its derivatives exceed this value. Only QUE‐3′,7 exceeds the size parameter (MW: 625.14 g/mol), the MW of QUE is 302.23 g/mol and for QUE‐3′α, QUE‐7α, and QUE‐3β is 463.08 g/mol and 464.38 g/mol, respectively. The other parameters are within the established range for all the molecules.

Plasma protein binding (PPB) and volume of distribution (VD) were considered to study distribution. The VD was higher for the QUE‐3′α, QUE‐3β, and QUE‐3′,7 derivatives with values of 0.895, 0.872, and 0.871 L/kg, respectively. Consistent with these results, the ideal percentage for PPB is <90%, observed for all derivatives, whereas QUE exceeds this value (95.49%), indicating that it is mostly bound to plasma proteins, especially albumin, whereas the derivatives are approximately 20% free in plasma and may enter tissues. This suggests that a significant amount of the glucosylated derivatives may be more readily available in the tissues, where they could potentially exert therapeutic effects.

A study assessing the pharmacokinetics of QUE and a glucuronide conjugate of the molecule, quercetin‐3‐*O*‐β‐glucuronide, following oral administration of 100 mg/kg of both compounds in rats, revealed that the AUC of the conjugate was 18 times higher than that of QUE [51]. Additionally, in the same study, following intravenous injection of 10 mg/kg, QUE and the glucuronide conjugate exhibited substantial tissue uptake in various organs, with concentrations measured as follows: kidney (409.2 ± 118.4 ng/g), liver (166.1 ± 52.9 ng/g), heart (97.7 ± 22.6 ng/g), and brain (5.8 ± 1.2 ng/g). Other studies have demonstrated that this conjugate accumulates in the brain and exhibits an inhibitory effect on the aggregation of amyloid‐β (Aβ) [52], indicating that this compound is capable of penetrating the BBB. This was also demonstrated in the study conducted by Ishisaka et al. [53], which showed the localization and anti‐inflammatory activity of quercetin‐3‐*O*‐β‐glucuronide in the brain.

The ADMETlab platform's pharmacokinetic predictions also include a detailed assessment of metabolism in relation to enzymes of the cytochrome P450 (CYP450) system, defining the probability of molecules being inhibitors/substrates (1) or non‐inhibitors/non‐substrates (0) of CYP's isoforms. QUE exhibited a high probability of inhibiting CYP1A2 (0.943) and a lower probability with respect to the other isoforms (CYP2C9 > CYP2D6 > CYP3A4 > CYP2C19). All glucosylated derivatives showed low probabilities of being inhibitors of any CYP isoform. Regarding being CYP substrates, QUE was more likely to be a substrate for CYP2C9 and CYP2D6, and less likely for the other isoforms (CYP1A2 > CYP3A4 > CYP2C19). The QUE‐3′α and QUE‐3β derivatives demonstrated the highest probability of being a substrate for CYP2C9 and CYP2D6, whereas the QUE‐3′,7 and QUE‐7 derivatives showed higher probabilities for CYP2D6. For the other isoforms, the probabilities were very low.

QUE and its metabolites undergo additional phases I and II metabolism when they reach the liver, and to facilitate their excretion through the bile and urine, they will follow specific pathways such as glucuronidation, sulfation, and methylation [54]. Glucuronide conjugates are the main existing forms of QUE in the bloodstream [55]. Phase I of metabolism includes oxidation, reduction, and hydrolysis reactions [56]. These reactions are mainly mediated by CYP450, and although these enzymes do not have a major influence on the general metabolism of flavonoids [56], it is known that these molecules can activate or inhibit CYP activity. CYP450 enzymes play an important role in the metabolism of various endogenous compounds, drugs, and other xenobiotics. CYP3A4 is involved in the biotransformation of more than 50% of orally administered drugs; in addition, CYP2C19 and CYP2D6 are also relevant enzymes in drug metabolism [57].

Increasing evidence suggests that QUE may interact with several xenobiotics. Through in silico analysis, QUE was identified as having a high probability of being a competitive inhibitor of CYP1A2 and lower probabilities of the CYP2C9 and CYP2D6 isoforms, as also identified in the work of Rastogi et al. [58]. In the study by Bedada et al. [59], researchers administered QUE and observed a significantly decreased CYP2C9‐mediated elimination of diclofenac in healthy human subjects. QUE inhibits different CYP enzymes and may increase the bioavailability of a wide variety of drugs, such as fexofenadine [60] and cyclosporine A in humans [61]. However, according to the in silico prediction, none of the derivatives showed any likelihood of being inhibitors of any of the CYP isoforms, which could enhance safety in cases of drug coadministration.

In terms of the likelihood of being substrates for CYP enzymes, both QUE and its derivatives exhibited probabilities of being substrates for the CYP2C9 and CYP2D6 isoforms, with QUE showing the highest probability among the compounds. Therefore, based on this prediction, it can be inferred that these molecules are likely to undergo metabolism specifically by these isoforms, potentially affecting the bioavailability of certain drugs that are also metabolized by these same isoforms, such as metoprolol and simvastatin [62].

In addition to potentially being substrates for these CYPs, the glucosylated derivatives were highly likely to be substrates for P‐glycoprotein (P‐gp), with the QUE‐3′,7 derivative showing the highest probability (0.988) and the QUE‐3β derivative the lowest probability among the derivatives (0.447). QUE and its derivatives have a very low probability as inhibitors of P‐gp, with scores ranging from 0.001 to 0.009. For an inhibitor and/or substrate, the score is 1, whereas a score of 0 indicates a non‐inhibitor or non‐substrate.

P‐gp is an important efflux pump that removes a variety of foreign substances and xenobiotics from cells [63]. When a substance is a substrate of P‐gp, its pharmacological activity can be affected, as P‐gp is expressed in many tissues, such as the BBB [63], influencing the permeability of substances into the brain. The application of P‐gp inhibitors or reversal agents allows for a more effective administration of molecules that are P‐gp substrates, targeting them to the brain simultaneously with these agents. This may overcome the removal of P‐gp substrates from the brain, increasing their concentration in the area of action and therefore potentiating the desired therapeutic effects [64]. In addition, glucosylated derivatives can lose the glucoside group through metabolic processes in the body, resulting in the formation of aglycone, and through in silico predictions, QUE showed a low probability of being a P‐gp substrate.

According to half‐life, in silico predictions showed that QUE had the highest probability of having a lifespan of less than 3 h in the body (0–0.3: T1/2 excellent > 3 h; 0.3–0.7: moderate; 0.7–1.0: T1/2 poor < 3 h). The output value represents the probability of having a T1/2 < 3 h, within the range of 0–1, followed by the QUE‐3β, QUE‐3′α, and QUE‐7α derivatives compared to the diglucosylated derivative, which showed a lower probability of having a half‐life of less than 3 h. Therefore, among the derivatives, the β derivative has the highest probability of having a half‐life of less than 3 h in the body.

Complementing these results, clearance rate, which represents the body's ability to remove a substance from the blood or plasma over time, in silico predictions indicated that QUE has a higher clearance rate (8.284 mL/min/kg), meaning that it is efficiently removed from the body compared to its glucosylated derivatives, which showed a lower clearance rate (<5 mL/min/kg), especially QUE‐3′,7 (1.325 mL/min/kg), being the β derivative with the highest clearance rate compared to the α derivatives. A lower clearance rate can be considered advantageous in certain contexts, particularly in the pharmaceutical and clinical fields [65]. In terms of prolonging therapeutic effects, a lower clearance rate and a longer half‐life can lead to prolonged exposure to the active substance, resulting in a longer lasting therapeutic effect. This may be desirable for drugs requiring sustained action, such as treatments for chronic diseases or to reduce the frequency of administration [66].

**FIGURE 4:**
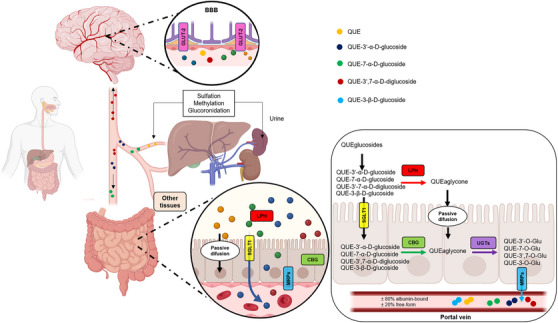
Pharmacokinetic profile of quercetin (QUE) and its glucosylated derivatives. Considering the oral administration of QUE and its derivatives, the lipophilic nature of QUE aglycone allows it to passively penetrate biological membranes, whereas glucosylated derivatives do so through specific transporters. In the apical membrane of enterocytes, some of these glucosides may undergo deglucosylation by enzymes such as LPH, generating QUE aglycone. Part of the glucosides that enter enterocytes may undergo deglucosylation by CBG. QUE aglycone undergoes biotransformation by UGTs, generating QUE conjugates that can passively pass through membranes or be transported by MRP2 to circulation. In plasma, approximately 80% of molecules bind to albumin. Upon reaching the liver, molecules will undergo Phase I (CYPs) and Phase II (glucuronidation, sulfation, and methylation) metabolism and may again be transported to circulation through MRPs located on the basolateral membrane of hepatocytes and distributed to tissues, potentially reaching the BBB, where there is high expression of GLUT‐2 transporters that can aid in the entry of glucosylated derivatives into the brain. CBG, β‐cytosolic glucosidases; GLUT‐2, glucose transporter‐2; LPH, lactase‐phlorizin hydrolase; MRP2, multiresistance protein 2; SGLT‐1, sodium‐dependent glucose transporter‐1; UGT, uridine‐5′‐diphosphate glucuronosyltransferases.

Overall, the glucosylated derivatives showed favorable pharmacokinetics in several aspects. They can be absorbed by the cells of the small intestine via specific transporters and enter the bloodstream (Figure 4). Once in the bloodstream, these derivatives can be distributed to various tissues and organs in the body, including the BBB. Through the in silico predictions obtained, it was possible to observe a higher VD compared to QUE, indicating that they can disperse more widely throughout the tissues. Additionally, QUE and its derivatives can undergo hepatic metabolism, where enzymes in the liver (such as CYP450) can modify their chemical structure, as well as undergo processes such as glucuronidation, sulfation, and methylation, which can alter their properties and biological activity. After metabolism in the liver, glucosylated derivatives and their metabolites can be excreted from the body, mainly through urine. The presence of sugar provides greater stability for the molecule, especially in the conditions of the gastrointestinal tract, thus offering protection against chemical and bacterial degradation [67]. It is important to note that the complete pharmacokinetic profile may vary depending on the specific structure of the derivative. These formulations were based on the profile of already established molecules, such as β‐glucosylated derivatives and QUE itself, as there were not many significant differences in the parameters analyzed for the α and β derivatives.

The analysis of toxicity parameters provided by ADMETlab is crucial to predict the safety and viability of a compound as a potential drug. By considering parameters such as hepatotoxicity, cardiotoxicity, mutagenicity, and others (Table 2), we can anticipate and mitigate potential adverse effects that a compound may cause in the body.

**TABLE 2:** Toxicity prediction of compounds.

Molecules	hERG blockers	Human hepatotoxicity	AMES toxicity	Rat oral acutetoxicity	FDAMDD
Quercetin	0.099	0.1	0.657	0.065	0.31
Quercetin‐3′‐α‐d‐glucoside	0.043	0.13	0.797	0.048	0.01
Quercetin‐3′,7‐α‐d‐diglucoside	0.019	0.083	0.723	0.034	0.002
Quercetin‐7‐α‐d‐glucoside	0.034	0.106	0.845	0.053	0.011
Quercetin‐3‐β‐d‐glucoside	0.101	0.137	0.837	0.052	0.012

*Note*: The output value is the probability of being toxic, within the range of 0–1. Empirical decision: 0–0.3: excellent; 0.3–0.7: medium; 0.7–1.0(++): poor. FDAMDD: maximum recommended daily dose.

In relation to the cardiotoxicity, all molecules showed a low probability of being blockers of the hERG protein (the value represents the probability of being a hERG blocker within the range of 0–1), which are channels responsible for controlling the flow of potassium ions across cell membranes, playing a crucial role in regulating the resting potential and duration of action potential in cardiac cells, meaning that QUE and its glucosylated derivatives have a low probability of being cardiotoxic [68]. Additionally, the molecules also showed a low probability of being hepatotoxic, with the QUE‐3′,7 derivative exhibiting the lowest probability compared to the others.

As for the acute oral toxicity parameter in rats (low toxicity, >500 mg/kg; Category 1: high toxicity; <500 mg/kg), all molecules presented low probability, especially the derivative QUE‐3′,7. Concerning the maximum recommended daily dose (FDAMDD), which provides an estimate of the toxic dose threshold of chemicals in humans, QUE exhibited the highest probability of being toxic (0.31) compared to its derivatives (Category 1: positive FDAMDD (+), ≤0.011 mmol/kg bw/day; Category 0: FDAMDD negative (−), >0.011 mmol/kg bw/day). QUE was the molecule that presented the lowest probability of being mutagenic (AMES test), compared to its derivatives, whereas the QUE‐7 derivative exhibited the highest probability. The AMES test is a mutagenicity assay that utilizes bacterial strains to assess the ability of a substance to cause reversible mutations in specific bacterial genes, indicating its genotoxic potential [69]. Although QUE has demonstrated the lowest mutagenic potential compared to its derivatives, many studies have evaluated the in vitro mutagenic potential of QUE when tested on bacterial strains. QUE exhibited high mutagenic activity, causing mutations and single‐strand breaks in DNA [70].

Studies examining genotoxic outcomes in mice and rats after oral administration of QUE and derivatives observed that the molecule did not exert any mutagenic or genotoxic properties in vivo [71]. Considering the previous effects presented for QUE and its derivatives, as well as their potential application for conditions of the nervous system, it was essential to evaluate their toxicity to cells. In this context, we investigated the impact that these substances can have on neural cells.

### 3.3 Comparative Analysis of Cytotoxic Effects of QUE and Its Glucosylated Derivatives

**FIGURE 5:**
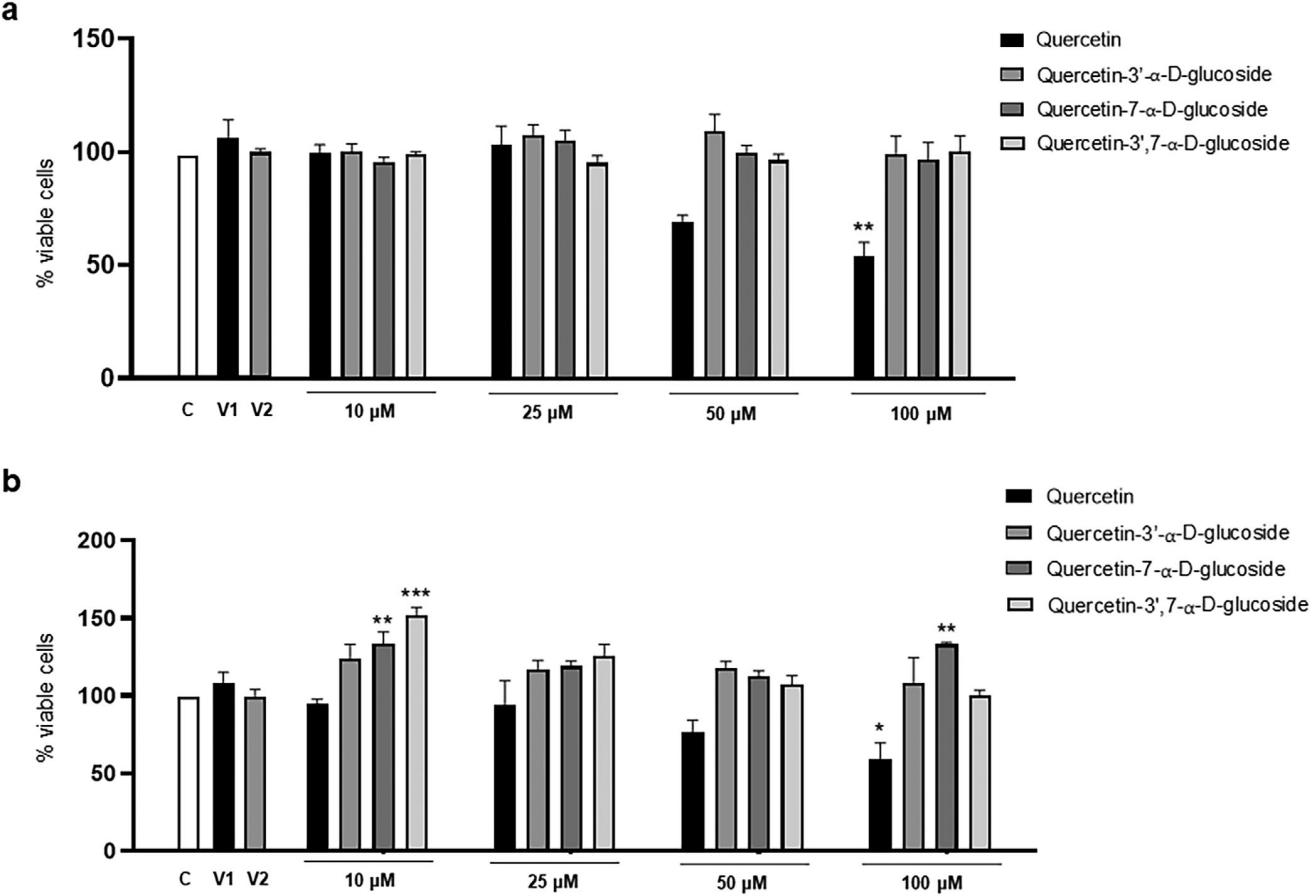
Effects of quercetin (QUE) and its derivatives on cell viability of C6 astroglioma (a) and neuroblastoma cells (SH‐SY5Y) (b). Cells were treated with QUE and the derivatives (10–100 µM) or vehicle (v1: DMSO 0.2% and v2: 0.6%) for 24 h. Cell viability was measured using MTT assay. Each bar represents the mean ± standard error of the mean of the percentage of viable cells in relation to the control group from three independent experiments performed in triplicate. *p < 0.05; **p < 0.01; *** p < 0.001 significantly different from control (one‐way ANOVA followed by Tukey post hoc test (a) and Dunnet (b)).

To investigate whether QUE and its derivatives exerted cytotoxicity on astroglioma (C6) and neuroblastoma (SH‐SY5Y) cells, the MTT viability assay was carried out. As shown in Figure 5, treatment with QUE (100 µM) for 24 h significantly decreased cell viability compared to the control group for both C6 (54.75% viability) and SH‐SY5Y (57.59% viability) cells, and the derivative Que‐3β exhibited the highest cytotoxicity among the molecules, reducing the viability of C6 cells starting at a concentration of 25 µM (60.78%) and SHSY‐5Y cells at a concentration of 100 µM (57.57%), compared to the control group. Interestingly, its α‐glucosylated forms showed no cytotoxicity at any of the concentrations evaluated during the 24 h. These results are consistent with those reported in the literature regarding QUE. Spencer et al. [72] also demonstrated that micromolar concentrations of QUE were neurotoxic to primary neurons and that its derivatives were much less neurotoxic than the original molecule. In the work of Nile et al. [73], the researchers also observed dose‐dependent cytotoxic effects of the Que‐3β derivative in HeLa cells, with cell growth inhibition occurring at concentrations higher than 10 µg/mL.

Although QUE and its derivative Que‐3β are molecules with many therapeutic properties, other studies in cell cultures have demonstrated their cytotoxic and pro‐oxidant effects at high concentrations. The concentration at which these molecules produce such negative effects depends on the cell type and exposure time, with concentrations above 100 µM particularly showing pro‐oxidant, apoptotic, antiproliferative, cytotoxic, and genotoxic activities [74]. Another study comparing the effects of QUE with glucosylated derivatives in PC12 cells observed that the derivatives did not exhibit cytotoxicity, with cell viability remaining above 80% at concentrations of 2.5–80 µM, whereas QUE significantly inhibited cell proliferation at concentrations above 20 µM. The authors concluded that glucosylated QUE exhibited greater safety and neuroprotection, as observed here for the α‐glucosylated derivatives. Although there are no comparative studies on the effects of β‐glucosylated and α‐glucosylated QUE derivatives, it is suggested that β‐glucosylated derivatives may be more cytotoxic than their α‐glucosylated counterparts. It is well‐established that glycosylation at different positions can significantly influence the biological activity of flavonoids.

## 4 Conclusion

Many flavonoids, despite presenting a wide range of beneficial biological activities for health, have pharmacokinetic limitations that hinder achieving adequate therapeutic concentrations in target tissues, especially in the brain. Faced with this issue, several studies have sought the structural optimization of natural molecules to improve their physicochemical properties and maximize their therapeutic potential. In this integrated approach, we evaluated the enzymatic potential of BaSP Q345F in modifying the chemical structure of QUE based on the transglucosylation reaction to produce glucosylated compounds. Three products were obtained, with the derivative quercetin‐3′‐α‐d‐glucoside being produced with a high yield rate.

These compounds were evaluated for their pharmacokinetic properties using computational tools capable of predicting and assessing these aspects quickly, economically, and efficiently. It was possible to obtain information on how the glucosylated derivatives of QUE are absorbed, distributed, metabolized, and excreted in the body. This helps us better understand how these compounds interact with the body and how their concentration varies over time. In general, the diglucosylated derivative presented the best parameters, including the highest VD, longer half‐life and lower clearance rate, higher percentage of being in its free form in plasma, and lower probability of presenting toxicity, and there were no significant differences between the α and β derivatives in these pharmacokinetic parameters.

In addition to these results, the analysis of in vitro cytotoxicity provided important comparative information about the toxicity of the α and β glucosylated derivatives compared to the original molecule. The α‐glucosylated derivatives did not show potential to cause damage in either cell type. However, the QUE and the Que‐3β derivative significantly reduced the viability of both cell types. Therefore, the presence of sugar in the α conformation in the chemical structure of a molecule can bring various benefits, not only providing greater solubility and stability, but also reducing its toxicity.

However, additional studies directly comparing the cytotoxic effects of α‐ and β‐glucosylated derivatives, exploring their interactions with cells and mechanisms of action, are of utmost importance. Furthermore, additional preclinical studies using in vivo models are crucial to better understand how these molecules behave in a complex organism. Potential interactions with other dietary components or physiological systems, as well as their transformation into various metabolites, can significantly influence their biological effects.

In summary, the results presented here, based on pharmacokinetic predictions and in vitro cytotoxicity assessments, may serve as a foundation for future in vitro studies aimed at understanding the biological effects and mechanisms of action of these molecules. They may also pave the way for future preclinical trials in animal models to more comprehensively investigate the therapeutic potential of these glucosylated QUE derivatives, particularly in CNS disorders, and to evaluate their safety and efficacy in a real clinical context.

## Conflicts of Interest

The authors declare no conflicts of interest.

## Supporting information



Supporting Information

## Data Availability

The data that support the findings of this study are available from the corresponding author upon reasonable request.
