# Supplementary material for: Pharmacokinetic Prediction and Cytotoxicity of New Quercetin Derivatives
Source: Chem Biodivers. 2025 May 9;22(8):e202500119. doi: 10.1002/cbdv.202500119 (PMC12351435; doi:10.1002/cbdv.202500119)
Supplement: Supplementary file 1 — Supporting Information [file CBDV-22-e202500119-s001.docx]

**Predictive pharmacokinetics and *in vitro* toxicity assessment of enzymatically produced glucosylated quercetin derivatives: an integrated approach.**

Supplementary material


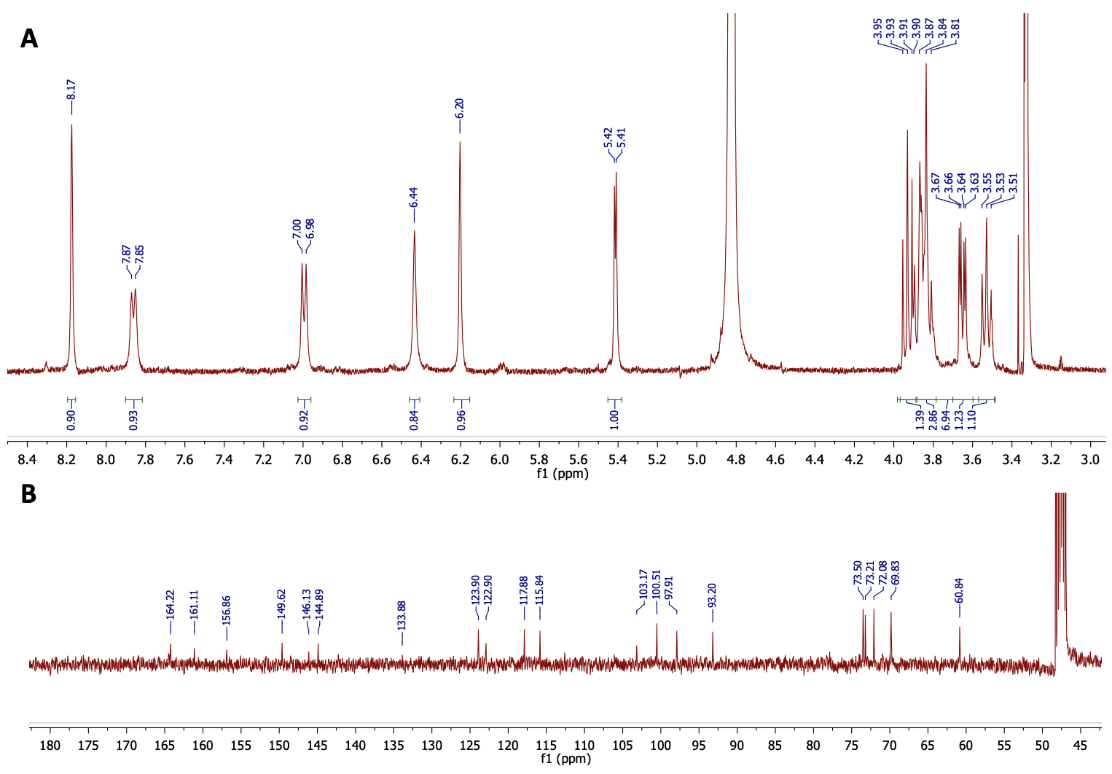


**Figure S1:** NMR spectra of quercetin-3’-O-α-D-glucoside (A) ^1^H spectrum; (B) ^13^C spectrum.


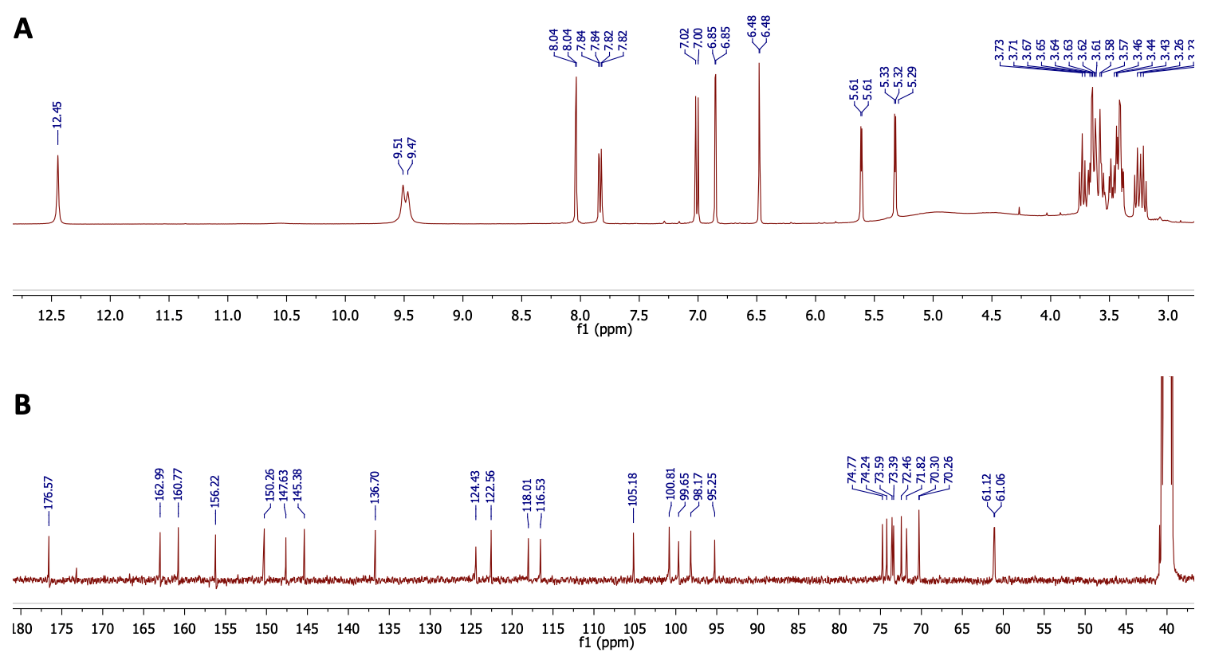


**Figure S2:** NMR spectra of quercetin-3’,7-O-α-D-diglucoside (A) ^1^H spectrum; (B) ^13^C spectrum.


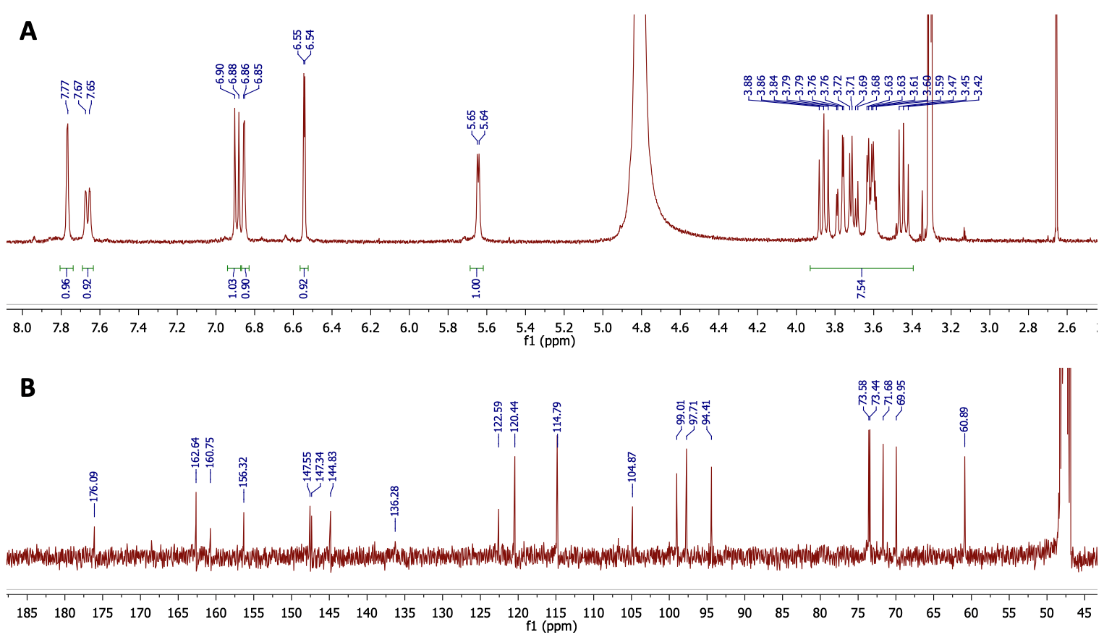


**Figure S3**: NMR spectra of quercetin-7-O-α-D-glucoside (A) ^1^H spectrum; (B) ^13^C spectrum.


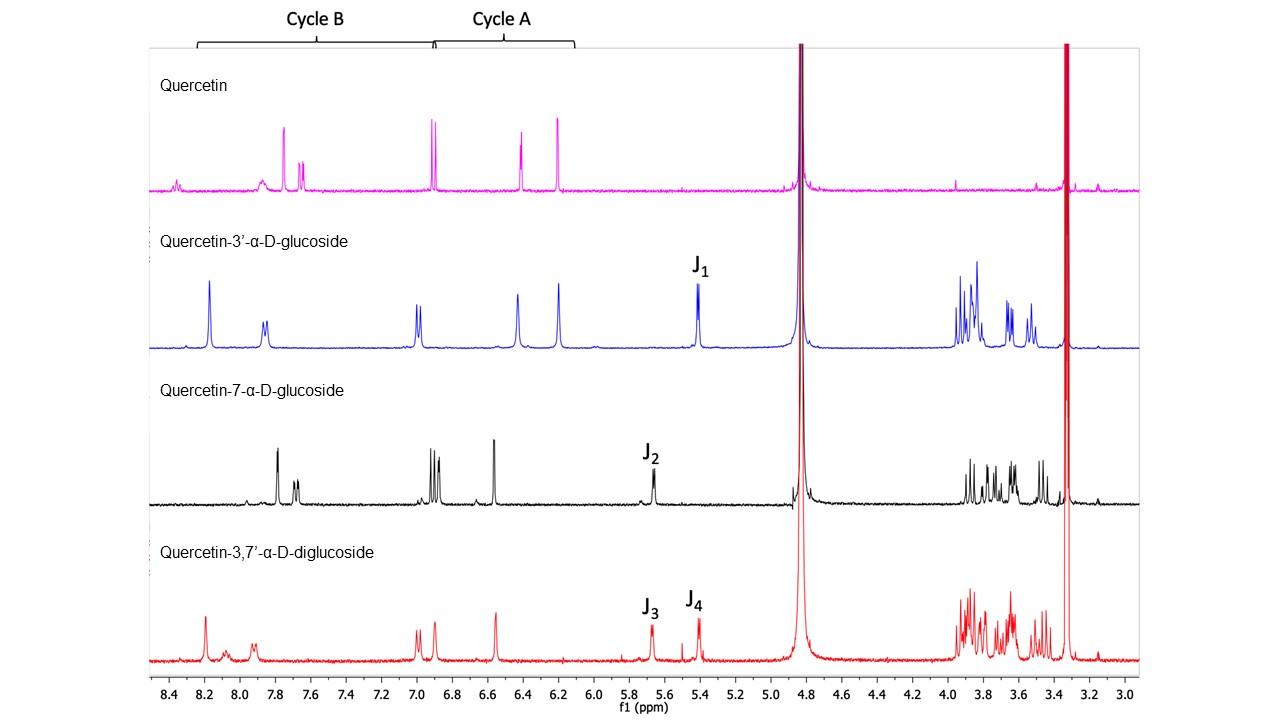


**Figure S4:** Comparison of the 1H NMR spectra of quercetin (magenta), quercetin-3’-⍺-D-glucoside (blue), quercetin-7-⍺-D-glucoside (black) and quercetin-3’,7-⍺-D-diglucoside (red). Coupling constant J1 = 3.6 Hz, J2 = 3.8 Hz, J3 = 3.3 Hz and J4 = 3.6 Hz.

**Table S1:** Validation of structures by mass spectrometry (negative ESI)

| Compound | Ion formula | Calculated m/z | Experimental m/z | Error (ppm) |
| --- | --- | --- | --- | --- |
| Quercetin-3’-⍺-D-glucoside | C_21_H_19_O_12_H^-^ [M-H]^-^ | 463.0877 | 463.0877 | 0.0 |
| Quercetin-7-⍺-D-glucoside | C_21_H_19_O_12_H^-^ [M-H]^-^ | 463.0877 | 463.0877 | 0.0 |
| Quercetin-3’,7-⍺-D-diglucoside | C_27_H_29_O_17_H^-^ [M-H]^-^ | 625.1405 | 625.1413 | 1.3 |
